# Tissue rheology in embryonic organization

**DOI:** 10.15252/embj.2019102497

**Published:** 2019-09-12

**Authors:** Nicoletta I Petridou, Carl‐Philipp Heisenberg

**Affiliations:** ^1^ Institute of Science and Technology Austria Klosterneuburg Austria

**Keywords:** embryonic development, morphogenesis, phase transitions, tissue material properties, tissue rheology, Development & Differentiation

## Abstract

Tissue morphogenesis in multicellular organisms is brought about by spatiotemporal coordination of mechanical and chemical signals. Extensive work on how mechanical forces together with the well‐established morphogen signalling pathways can actively shape living tissues has revealed evolutionary conserved mechanochemical features of embryonic development. More recently, attention has been drawn to the description of tissue material properties and how they can influence certain morphogenetic processes. Interestingly, besides the role of tissue material properties in determining how much tissues deform in response to force application, there is increasing theoretical and experimental evidence, suggesting that tissue material properties can abruptly and drastically change in development. These changes resemble phase transitions, pointing at the intriguing possibility that important morphogenetic processes in development, such as symmetry breaking and self‐organization, might be mediated by tissue phase transitions. In this review, we summarize recent findings on the regulation and role of tissue material properties in the context of the developing embryo. We posit that abrupt changes of tissue rheological properties may have important implications in maintaining the balance between robustness and adaptability during embryonic development.

**Glossary**
Adaptability: The ability of a system to adapt itself into a new state upon perturbations
Control parameter: A thermodynamic variable of a system that when acquiring a critical value causes a discontinuity to the order parameter
Critical point: The end point of a phase equilibrium curve
Criticality: The vicinity to the critical point of a phase transition
Elasticity: Ability of a material to recover its original configuration (a reference state that the system remembers) when a given applied stress is released
Fluidity: Inverse of viscosity, the ability of a material to flow under a given applied force
Fluidization: A change from a static solid‐like state to a dynamic fluid‐like state
Glassy materials: Materials with the mechanical properties of solids but microscopically exhibiting the disordered structure of a liquid
Jamming: Divergence of the viscosity of a material (i.e. polymers, granular materials, glasses, foams) with increasing particle density into an amorphous solid‐like state
Order parameter: Measure of the degree of order, which exists in the one phase and disappears in the other upon a phase transition
Phase transition: Abrupt change of a phase (solid, liquid, gas) when a control parameter is infinitesimally modified
Power law: The functional relationship in which a relative change in one quantity gives rise to a proportional relative change in the other quantity, independently of the initial size of those quantities
Rheology: The study of how materials with both solid and fluid characteristics react under forces
Rigidity: The ability of a material to withstand deformation under mechanical stress
Robustness: The resistance of an initial stable configuration of a system to changes upon perturbations
Scale invariance: Features of objects that do not change when scales of other variables are multiplied by a common factor
Solidification: A phase change that makes a material solid
Stiffness/Elastic or Young's modulus: Modulus of elasticity, the ratio of stress to strain in the elastic region. Unit: N/m
Strain: Measure of the deformation of a material in response to an applied stress
Universality: At a critical point, systems acquire characteristics, which are shared with many other systems, irrespective of many microscopic details and variables
Viscosity: Measure of the stress required for a material to flow at a given velocity. Unit: Pa.s
Wetting: The ability of a liquid to maintain contact with a solid surface due to the force balance between adhesive and cohesive forces
Yield stress: The maximal mechanical stress that a material can sustain in a solid‐like state before starting to re‐organize and exhibit a plastic or permanent deformation

## Introduction

How the single‐cell totipotent zygote can transform into a fully functional multicellular organism is a long‐standing unresolved problem at the interface of developmental biology, physics and evolution. The seminal works of Alan Turing “The chemical basis of morphogenesis” (Turing, 1952) and D'Arcy Thomson “On growth and form” (Thompson, 1917) have set the basis for our current understanding of how the spatial and temporal coordination of interdependent biochemical and mechanical signals control organismal development.

The developing embryo initially consists of a seemingly uniform cell mass, in which symmetry needs to be broken or existing asymmetries to be amplified (Blum *et al*, 2014; Zhang & Hiiragi, 2018). Chemical signals, also called morphogens, can prime certain cells within this mass to acquire a distinct fate and behaviour. Often, these fate specification processes involve reaction–diffusion systems of activators and inhibitors triggering symmetry breaking and embryo patterning (Wartlick *et al*, 2009; Briscoe & Small, 2015). Mechanical signals such as applied forces and geometrical constraints, not only directly affect cell shape and position within the embryo, but can also be “read” or “sensed” by molecular components, typically belonging to the cytoskeletal and adhesion apparatus. These mechanosensitive responses lead to the transformation of mechanical into biochemical signals that in turn can influence cell specification and morphogenesis (Heisenberg & Bellaïche, 2013; Petridou *et al*, 2017). Mechanical and chemical signals can also form a feedback loop, thereby linking the mechanical processes underlying cell and tissue morphogenesis with the gene regulatory pathways determining cell fate specification (Mercker *et al*, 2016; Barone *et al*, 2017; Hannezo & Heisenberg, 2019; Xia *et al*, 2019).

**Figure 1:**
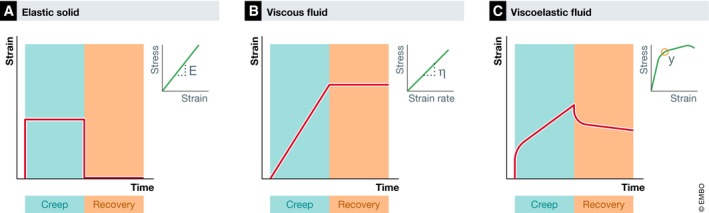
Identification of material properties through stress‐relaxation tests Strain (ε)—time, stress (σ)—strain (ε) and stress (σ)—strain rate (ε%) plots of the material response upon application (creep, green shaded box) and release (recovery, orange shaded box) of mechanical force (A–C).
An elastic solid material displays proportionality between stress and strain. It deforms immediately upon stress application and returns to the initial shape once the force is removed. From the stress–strain plot, the elastic modulus (E) can be calculated as: σ = Eε.A viscous fluid material displays proportionality between stress and strain rate. It deforms gradually upon stress application and its strain increases over time irreversibly. Once the force is removed, due to energy dissipation, the acquired new shape is retained. From the stress–strain rate plot, viscosity (η) can be calculated as: σ = ηε%.A viscoelastic material displays at short timescale an immediate elastic deformation, which is followed by a viscous flow at long timescales during mechanical force application. When force is removed, some deformation is quickly recovered due to the elastic nature of the material and the rest of the deformation then gradually decreases either partially (viscoelastic fluid) or completely (viscoelastic solid). From the stress–strain plot, the yield stress (y) can be identified at the point where the elastic deformation stops and the viscous deformations begins (circle).Abbreviations: ε, strain; σ, stress; E, elastic modulus; ε%, strain rate; η, viscosity; y, yield stress.

Embryo morphogenesis not only depends on the forces generated, transmitted and sensed within the organism, but also on the material properties of the constituent cells and tissues (Davidson, 2011). Generally, the material or rheological properties of cells and tissues determine to what extent they deform in response to extrinsic or intrinsically generated forces (Lecuit *et al*, 2011). The most common experimental tool to measure the rheological properties of a material is the “creep and recovery” test, where the *strain* or deformation of a material is monitored in response to force application and removal (Gutierrez‐Lemini, 2014) (Glossary; Fig 1). The deformation of a material in response to changes in stress can exhibit characteristics of solids and/or fluids (Özkaya *et al*, 1991). A solid material exhibits an elastic deformation, where it deforms as long as the force is applied and returns to its original shape when the force is removed (Fig 1A). A fluid‐like material exhibits a viscous deformation, where its deformation increases over time and it is irreversible when the force is removed (Fig 1B). By analysing the stress–strain relationship over time, several rheological characteristics of a material, such as *stiffness/elastic modulus*,* viscosity/fluidity* and *yield stress,* can be obtained (Glossary; Fig 1) (Özkaya *et al*, 1991; Bonn *et al*, 2017). In pioneering studies by Steinberg and colleagues, such stress‐relaxation experiments using a *parallel plate compression* apparatus (Box 1) on isolated spherical cell aggregates from living embryonic tissues revealed that those tissues exhibit both viscous and elastic properties with an elastic response dominating at short time scales and a viscous response at longer time scales (Fig 1C) (Forgacs *et al*, 1998). Similar to the viscoelastic behaviour of cell–cell contacts (Clément *et al*, 2017), these tissue‐scale viscoelastic properties could confer both *robustness* and plasticity to tissues by allowing them not only to maintain integrity when challenged by short‐term mechanical perturbations (solid‐like characteristic), but to also permanently change their shape when exposed to forces at longer time scales during embryonic development (fluid‐like characteristic). Moreover, given that morphogenesis, fate specification and motion of cells are influenced by the *rheology* of their microenvironment (Engler *et al*, 2006; Rozario *et al*, 2009; Rozario & DeSimone, 2010; Trichet *et al*, 2012; Petridou *et al*, 2013; Bonnans *et al*, 2014), this suggests that the specific viscous and elastic properties are an important factor influencing the morphogenetic capacity of developing tissues. However, the study of tissue rheology in development is still in its infancy, partially due to the lack of suitable techniques in systematically determining spatiotemporal variations in tissue rheological properties within the developing organism, the development of which has only recently gained momentum (summarized in Box 1 and reviewed in Campàs 2016).

**Box 1: Biophysical tools measuring embryonic tissue rheology**

*Cell/tissue force spectroscopy*: Cell/tissue force spectroscopy is an Atomic Force Microscopy (AFM) application used for measuring material properties of cells and tissues. It records the bending of a cantilever with known mechanical properties that approaches and contacts the surface of a cell or tissue at a defined speed (Gautier *et al*, 2015; Haase & Pelling, 2015; Krieg *et al*, 2019). For probing large‐scale material properties within embryonic tissues, typically a large bead is attached to the AFM cantilever in order to avoid probing local cell heterogeneities. Cell/tissue force spectroscopy has been used, e.g., for probing the elasticity of *Xenopus* head mesoderm (Barriga *et al*, 2018), chick embryonic digestive tract (Chevalier *et al*, 2016) and of the basement membrane of the *Drosophila* egg chamber (Crest *et al*, 2017). Viscous moduli can also be extracted using this method by maintaining a constant cantilever displacement while in contact with the cell/tissue over time (Mathur *et al*, 2001). The major limitation of this method is that it is difficult to decouple surface from deep cell/tissue properties.

*Brillouin Microscopy (BM)*: BM probes the viscoelastic properties of a material via light scattering. A visible or monochromatic laser light is scattered upon interaction with acoustic waves that are spontaneously induced by density fluctuations within a sample (phonons, microscopic sounds waves). This generates a Brillouin spectrum where the scattered light displays a frequency shift compared to the illumination light, from which the longitudinal modulus can be measured if the refractive index and density of the material is known. The longitudinal modulus serves as an approximate for rigidity since it is higher for more rigid materials and lower for less rigid materials. In addition, from the linewidth of the Brillouin peaks the viscosity of the tissue can be obtained (preprint: Prevedel *et al*, 2019). BM has been used to map 3D ECM stiffness of the notochord in zebrafish embryos (Bevilacqua *et al*, 2019). The main advantage of this technique compared with other techniques is that it can be used to map tissues in 3D and that it is non‐invasive, as it does not require any contact to the sample. However, in order to obtain accurate measurements, the density and refractive index of the material needs to be known, which is often not the case for embryonic tissues. Moreover, the longitudinal modulus does not always correlate with the elastic modulus of the material (e.g. in highly hydrated materials) (Wu *et al*, 2018) and thus can only be used as an approximate measure of tissue elasticity for certain tissues.

*Explant Shape Analysis (ESA)*: ESA is a simple technique, which allows measuring the relationship of surface tension (TST) and viscoelastic properties of tissues *ex vivo*. The tissue of interest is dissected from the embryo and its deformation is monitored in a culture dish. Assuming that it behaves as a liquid drop, its rate of deformation is driven by its TST and resisted by its viscosity (Phillips & Steinberg, 1978). This technique and adaptations of it (e.g. explant fusion assay, axisymmetric drop shape analysis, centrifugation) has been widely used for embryonic tissues such as the zebrafish blastula (Morita *et al*, 2017), the *Xenopus* gastrula (Kalantarian *et al*, 2009; Luu *et al*, 2011; David *et al*, 2014) and several chick organ structures (Phillips & Steinberg, 1969). ESA is a powerful technique for global scale tissue mechanical measurements. However, it does not allow local measurements to probe potential tissue rheological heterogeneity. In addition, the results do not necessarily correspond to the *in vivo* situation, since the tissue *ex vivo* might display different mechanical properties than *in vivo*.

*Ferrofluid oil Droplets (FDs)*: FDs are inserted between the cells of a tissue, and their initial spherical shape is deformed generating a local force dipole upon the application of an external uniform directional magnetic field (Serwane *et al*, 2017). Imaging the deformation of the droplet shape during the application and removal of the magnetic force, the local tissue mechanical properties (viscosity, elasticity and yield stress) in a small neighbourhood surrounding the FD can be quantified, as demonstrated in the somitic mesoderm of zebrafish embryos (Serwane *et al*, 2017; Mongera *et al*, 2018). A great advantage of this technique is that both material properties and cellular topology can be analysed within the same embryo, allowing real‐time systematic analysis of their relationship. The local limitation of the measurements can be bypassed by inserting several droplets within the same tissue in order to extract larger‐scale measurements of the material properties. A major challenge is that tissues of different rigidity require droplets of different ferrofluid concentrations, given that the ferrofluid concentration within the droplet determines the maximal magnetic stress that can be applied by the droplet on the tissue.

*Magnetic Beads (MBs)*: MBs have been used for performing “creep and recovery” tests by inserting magnetic beads into the tissue of interest and—typically by using a magnetic tweezer—applying a directional controlled force that moves the bead within the tissue. By monitoring the extend of the bead movement during the application and removal of the magnetic force, the viscoelastic properties of the surrounding tissue can be quantified using simple mechanical models (Savin *et al*, 2011). MBs‐based methodologies have been used to measure viscoelastic properties of embryonic tissues such as elasticity of the trophectoderm and inner cell mass of the mouse blastocyst (Wang *et al*, 2018) and mouse limb bud (preprint: Zhu *et al*, 2018) and Young modulus, shear viscosity and bulk viscosity of the *Drosophila* blastoderm during cellularization (Doubrovinski *et al*, 2017; D'Angelo *et al*, 2019). Similar to the FDs, measuring tissue rheology through MBs at larger scales requires insertion of multiple beads within the tissue and the application of a uniform and strong magnetic field, a non‐trivial approach when, e.g., using magnetic tweezers.

*Micropipette Aspiration (MPA)*: MPA is used to perform “creep and recovery” tests on cells/tissues by applying a negative constant pressure, which is greater than the critical pressure of the sample, using a glass micropipette, thereby inducing a deformation/flow of the tissue in the micropipette at a rate dependent on the tissue resistance to deformation. During the application and release of the pressure, the deformation of the tissue over time is monitored, from which the surface tension, elastic modulus and viscosity can be quantified (Guevorkian *et al*, 2010; Guevorkian & Maître, 2017). This technique has been widely used to measure tissue viscoelasticity in several embryonic tissues, such as the *Xenopus* gastrula (von Dassow & Davidson, 2009; von Dassow *et al*, 2011), the developing chicken heart and brain (Majkut *et al*, 2013) and the zebrafish blastula (Petridou *et al*, 2019). While this technique has been successfully used to measure the viscosity of deep tissues (Petridou *et al*, 2019), decoupling surface from deep properties of the tissue requires selectively aspirating these different tissue fractions, a technically demanding task. MPA also does not allow very fine mapping of tissue material properties to reveal potential heterogeneities in the tissue.

*Parallel Plate Compression (PPC)*: During PPC, the tissue of interest is compressed at a fixed strain between two parallel rigid plates, and the force exerted by the tissue onto the plates is monitored upon the compression. Typically, tissues exhibit a viscoelastic relaxation until the compressive force reaches an equilibrium, which can be used to calculate the elastic modulus and viscosity of the tissue using standard mechanical models (Foty *et al*, 1996; Forgacs *et al*, 1998). PPC has been used to probe viscoelasticity of explanted embryonic tissues from chick (Forgacs *et al*, 1998) and zebrafish (Schoetz *et al*, 2013). Similar to the ESA, PPC provides information of global but not local tissue properties in culture, which might differ from their properties *in vivo*.

While most data so far support a permissive role of tissue material properties in tissue morphogenesis (Shook *et al*, 2018; Duda *et al*, 2019; Iyer *et al*, 2019), recent work also suggests that developing tissues can actively undergo pronounced changes in their material properties, modulating tissue shape changes (Petridou *et al*, 2019) and triggering other processes, such as cell migration (Barriga *et al*, 2018). In some cases, these changes can be abrupt, thereby resembling *phase transitions* (i.e. loss or gain of rigidity; Glossary) (Schoetz *et al*, 2013; Bi *et al*, 2015, 2016; Park *et al*, 2015), which recently have been speculated to represent important regulatory mechanisms in development (Mongera *et al*, 2018; Petridou *et al*, 2019). Phase transitions between ordered and disordered states occur at *critical points*, which might allow the embryo to balance between robustness against perturbations (order) and *adaptability* to changing conditions (disorder) (Hidalgo *et al*, 2013) (Glossary). The appearance of potential tissue phase transitions within the developing embryo suggests an active regulatory role of tissue rheology in embryo morphogenesis. It also opens new directions in understanding embryo development based on the principle of *criticality*—being close to a critical point where phase transitions occur—which might represent a key step for symmetry breaking and self‐organization in development.

In this review, we summarize and discuss experimental and theoretical work on the characterization and function of tissue‐scale rheological properties during embryonic development. We will first introduce the different rheological states a tissue can exhibit and how these states are defined by certain tissue architecture features. We will then summarize and discuss recent findings on the mechanochemical signals regulating tissue rheological dynamics. Lastly, we will discuss the possibility of whether and how tissue phase transitions occur in development, and which function they might have therein.

## Tissue rheology defined by cellular topology

Within a developing organism, different tissues can display different cellular features, such as cell shape, packing and motion (Hagios *et al*, 1998; Paluch & Heisenberg, 2009). Such features can be informative of the material properties and thus the phase state of a tissue, which is usually categorized as “fluid‐like” or “solid‐like” states, but also in some cases as a “gas‐like” state. When a tissue transits from a gas to a fluid‐like state, or from a fluid to a solid‐like state, its deformability decreases. From theoretical work and *in vitro* experiments, specific cellular parameters (*control parameters*, Glossary) have been identified that not only quantitatively characterize the phase state of a tissue, but upon fine‐tuning and regulation can also trigger abrupt transitions between the different phase states (Angelini *et al*, 2011; Bi *et al*, 2015, 2016; Park *et al*, 2015; Merkel & Manning, 2018). Such cellular parameters can thus be used to provide a universal description of the rheological state of a tissue and reveal the biological mechanisms underlying this state. Moreover, deciphering the relationship between the rheological state of a tissue and certain associated cellular processes can be instrumental in understanding morphogenetic processes in development and disease. For example, *in vivo* collective motion of germ layers relies on regulated tissue viscoelasticity (Moore *et al*, 1995; Barriga *et al*, 2018), and tumour growth, spreading and metastasis were associated with tissue stiffness and solid‐to‐fluid transitions (Oswald *et al*, 2017). In the following section, we will discuss the theoretical framework of how the cellular organization relates to the rheological state of a tissue, and how such framework can be applied for understanding how embryonic tissues acquire their specific rheological pattern.

**Figure 2:**
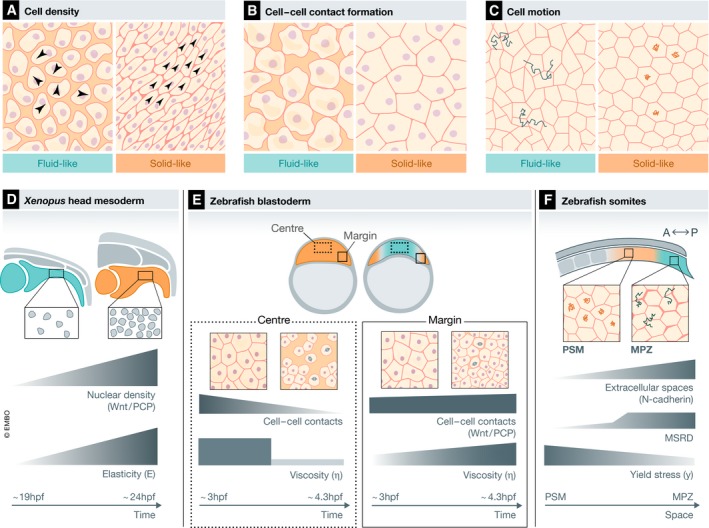
Cellular topology and tissue viscoelasticity in embryonic development Schematic diagrams of the cellular topology of fluid‐like (blue shaded boxes) and solid‐like (orange shaded boxes) tissues as defined by different cellular parameters (A–C) and representative examples of such tissues in the developing embryo (D–E).
At low cell density, cells display high and random cell motility (large black arrowheads, left panel) and low attachment to their neighbours, characteristic for a fluid‐like tissue. At high cell density, cell motion slows down and becomes more coordinated and directional (small black arrowheads, right panel), characteristic for a solid‐like tissue. A fluid‐to‐solid phase change for tissues that reach a critical density has been described as a jamming transition (Szabó *et al*, 2006; Sadati *et al*, 2013).At constant density, a tissue can acquire a fluid‐like state when its cells have small and weak cell–cell contacts (left panel) and a solid‐like state when its cells have large, mature and strong cell–cell contacts (right panel). Changes between solid and fluid phases for tissues that reach a critical adhesion value have been described as rigidity transitions or solidification (for fluid‐to‐solid) and fluidization (for solid‐to‐fluid) (Garcia *et al*, 2015).A confluent tissue (without interstitial gaps) with cells displaying asymmetric cell shapes and diffusive motion (exemplary trajectories in blue) is in a fluid‐like state (left panel), while a confluent tissue consisting of cells with highly symmetric cell shape and caged motion (exemplary trajectories in orange) is in a solid‐like state (right panel). A fluid‐to‐solid phase change of a tissue that reaches a critical value of cortical tension, adhesion and diffusive motility has been described as a density‐independent rigidity transition (Bi *et al*, 2015, 2016; Yang *et al*, 2017; Merkel & Manning, 2018).Schematics of *Xenopus* head mesoderm morphogenesis from the end of gastrulation (˜19 hpf) until the end of neurulation (˜24 hpf). At the end of gastrulation, the mesoderm displays low nuclear density (left panel), which gradually increases until the end of neurulation (right panel). This increase corresponds to a gradual increase in tissue elasticity (E) as measured by *in vivo* AFM. The increase in nuclear density and apparent elasticity depends on non‐canonical Wnt signalling (Barriga *et al*, 2018). The blue and orange tissues correspond to the rheological state as defined in (A).Schematics of the development of the zebrafish early embryo from blastula (˜3 hpf) to dome stage (˜4.3 hpf). At blastula, the blastoderm consists of highly adhesive cells with many, large and long‐lived cell–cell contacts and small interstitial gaps between each other (left panel) and displays uniform viscosity (η). Until the onset of doming (˜4 hpf), cells in the blastoderm centre gradually detach from each other leading to a gradual reduction in the number, size and longevity of cell–cell contacts and an increase in the size of interstitial gaps between these cells (dashed box, right panel). This eventually leads to an abrupt fluidization of the central blastoderm at the onset of doming, as measured by MPA. Non‐canonical Wnt signalling blocks tissue fluidization in the blastoderm margin (Petridou *et al*, 2019). The blue and orange tissues correspond to the rheological state as defined in (B).Schematics of the zebrafish body axis at 10‐somite stage (˜14 hpf). The body axis displays an anterior to posterior gradual decrease in the yield stress (y), with anterior tissues (PSM) being more rigid that the posterior tissues (MPZ), as measured by FDs. This is accompanied by an inverse gradient in the amount of extracellular spaces and random motion (MSRD). The establishment of the extracellular space and yield stress gradients depends on the function of N‐cadherin (Mongera *et al*, 2018). The blue and orange tissues correspond to the rheological state as defined in (C).Data information: Abbreviations: hpf, hours postfertilization; A‐P, anterior–posterior; PSM, presomitic mesoderm; MPZ, mesodermal progenitor zone; MSRD, mean squared relative displacement; AFM, Atomic Force Microscopy; MPA, micropipette aspiration; FDs, ferrofluid droplets.

In general, solid‐like tissues are often associated with high cell density, symmetrical cell shapes and persistent cell motion. Fluid‐like tissues, in contrast, are typically associated with low cell density, asymmetry in cell shape, and random cell motion and/or frequent cell rearrangements (Szabó *et al*, 2006; Angelini *et al*, 2011; Bi *et al*, 2015, 2016; Yang *et al*, 2017; Merkel & Manning, 2018). Finally, gas‐like tissues were linked with individual cell motility (Douezan *et al*, 2011). More specifically, models from statistical mechanics predict that cell monolayers below confluency level (particulate matter) display a disordered/fluid‐like state (Fig 2A). When cell density is increased in those monolayers, e.g. due to cell proliferation, cell motion in crowded areas slows down, cell cohesion increases, and the cell layer acquires an ordered/solid‐like state (Szabó *et al*, 2006; Angelini *et al*, 2011; Sadati *et al*, 2013) (Fig 2A). Moreover, in monolayers of high cell density (confluent matter), changes in cell adhesion can influence the rheological state of the cell layer: modelling cells in dense monolayers as self‐propelled interacting particles predicts that maturation and strengthening of both cell–cell and cell–substrate adhesion induces a solid‐like monolayer (Garcia *et al*, 2015) (Fig 2B). In contrast, when using vertex models, where cell shape is determined by a combination of cell–cell adhesive stresses and cortical tension, increased cell cohesion in confluent epithelial monolayers was predicted to induce a liquid‐like phase (Bi *et al*, 2015; Park *et al*, 2015). Subsequent work suggested that cell geometry might be a more reliable parameter determining the phase state of confluent cell layers and tissues, as it also provides information not only about cell cohesion but also the mobility state of the individual cells (Yang *et al*, 2017; Merkel & Manning, 2018). For instance, asymmetric cell shapes with long cell–cell contacts offer more degrees of freedom to promote cell rearrangements compared to isotropic cell shapes (Bi *et al*, 2016) (Fig 2C).

Testing those theoretical predictions in experiments revealed that the above identified cellular control parameters can indeed accurately describe the rheological properties of embryonic tissues during morphogenesis. For instance, there is experimental evidence linking cell packing/density to embryonic tissue *rigidity*. By monitoring *Xenopus* embryos *in vivo*, a gradual increase in cell density within the head mesoderm was associated with a gradual increase in the apparent *elasticity* of this tissue when probed by Atomic Force Microscopy (AFM, Glossary and Box 1; Fig 2D) (Barriga *et al*, 2018). There is also increasing evidence linking cell motion/rearrangements to tissue viscosity. In the zebrafish and chicken presomitic mesoderm (PSM), for instance, different cell flows and rearrangement rates were attributed to different fluid‐like states of the tissue (Bénazéraf *et al*, 2010; Lawton *et al*, 2013). In zebrafish, these observations were further confirmed by the use of *ferromagnetic oil droplets* (FDs) to directly measure tissue viscosity within the PSM (Box 1; Fig 2F), pointing at the possibility that the mesoderm transits from a fluid into a more solid‐like state during maturation along its anterior–posterior axis (Serwane *et al*, 2017; Mongera *et al*, 2018). A similar link between tissue fluidity and cell rearrangements is also found in several other embryonic tissues. For example, the viscosity of *Xenopus* embryonic tissues, when measured by *explant shape analysis* (ESA, Box 1), appears to be linearly correlated with the surface tension of the tissue, a relationship defined by the rate of cell rearrangements within the tissue (David *et al*, 2014). Moreover, during avian and zebrafish gastrulation, extensive cell rearrangements are thought to maintain the tissue in a fluidic state (Fig 2E) (Firmino *et al*, 2016; preprint: Saadaoui *et al*, 2018; Petridou *et al*, 2019). In addition to cell density and rearrangements, cell geometry also serves as a reliable readout of embryonic tissue fluidity. By analysing variations in cell shape within the forming ventral furrow in *Drosophila*, solid‐like areas within the furrow were shown to correspond to less elongated and variable cell shapes, while more fluid‐like areas exhibit longer and more variable cell shape that allows more frequent cell rearrangements (Atia *et al*, 2018). Similarly, in the *Drosophila* pupal wing epithelium, the rate of cell elongation over time represents a reliable readout of the viscoelastic behaviour of the tissue during wing deformation (Iyer *et al*, 2019).

While the theoretical considerations and experimental observations discussed above clearly show a link between certain cellular parameters, such as cell density, rearrangements and shape, with the tissue phase state and its resulting morphogenetic capacity, remarkably little is yet known on how these parameters are spatiotemporally regulated within the developing organism. Moreover, whether their regulation can change the phase state of a tissue decisively influencing its morphogenetic activity is not clear. In the following section, we will discuss recent evidence supporting a key role of regulated changes in tissue rheology affecting tissue morphogenesis, and summarize new findings on the mechanochemical regulators of tissue rheology within the developing embryo.

## Regulation of tissue rheology by morphogenetic signals

Recent studies suggested that key morphogenetic processes during vertebrate gastrulation, such as body axis elongation and convergence and extension movements, require dynamic changes in tissue stiffness and viscosity. Moreover, such rheological changes appear to be conserved between different species and regulated by similar mechanisms.

It has been known for long that mesoderm involution and convergent extension movements in *Xenopus* rely on selective tissue stiffening of the involuting marginal zone. Through stress‐relaxation experiments on the involuting mesodermal tissue before and after the onset of gastrulation, it was found that the tissue elastic modulus strongly increases upon internalization, pointing to the possibility that this increase in tissue stiffness may help in maintaining a straight body axis during subsequent gastrulation (Moore *et al*, 1995). Furthermore, by performing uniaxial tensile stress‐relaxation experiments on explanted mesodermal tissues from *Xenopus* embryos at the onset of neurulation, it was found that mesoderm stiffness increases, which might be important for allowing long‐range transmission of the convergent forces within the mesoderm (Shook *et al*, 2018). The cellular and molecular mechanisms underlying the observed mesoderm stiffness increase are not yet known, but it is conceivable that key signalling pathways controlling vertebrate gastrulation movements might be involved. In particular, the Wnt/PCP pathway might control mesoderm stiffness through its previously reported activities on regulating cell adhesion, extracellular matrix (ECM) deposition and collective cell motion (Wallingford *et al*, 2002; Tada & Heisenberg, 2012). Consistent with this possibility, the stiffness of the *Xenopus* head mesoderm, measured by AFM *in vivo*, was recently shown to gradually increase during neurulation, an effect that depends on intact Wnt/PCP signalling and cell contractility, affecting cell density and packing (Fig 2D) (Barriga *et al*, 2018). The increase in head mesoderm stiffness was further shown to be critical for neural crest cell migration, with neural crest cells mechanosensing mesoderm substrate stiffness in order to undergo epithelial‐to‐mesenchymal transition (EMT) and subsequent directed migration (Barriga *et al*, 2018). Interestingly, similar observations were made in studies on the development of the early mouse limb bud, where a Wnt5a gradient leads to a graded ECM deposition within the bud, which again translates into a gradient in limb mesodermal stiffness required for proper mesoderm migration (preprint: Zhu *et al*, 2018). This points at the intriguing possibility that the crosstalk between Wnt/PCP‐dependent regulation of mesoderm tissue stiffness and directed cell migration may represent a conserved regulatory mechanism in tissue morphogenesis.

An important role of Wnt/PCP signalling in regulating tissue rigidity was also recently demonstrated within the zebrafish early gastrula. Zebrafish morphogenesis begins with the process of doming, where the blastoderm spreads over the yolk cell, resulting in extensive thinning in its central region (Lepage & Bruce, 2010; Bruce, 2016; Morita *et al*, 2017). By measuring blastoderm viscosity during doming through *micropipette aspiration* (MPA; Box 1), the central region was found to undergo a sudden and drastic drop in viscosity akin a tissue *fluidization* (Glossary), whereas the marginal blastoderm, giving rise to the presumptive mesoderm (Warga & Kimmel, 1990; Warga & Kane, 2003), did not fluidize (Petridou *et al*, 2019) (Fig 2E). Fluidization of the blastoderm centre is thought to be the consequence of loss of cell–cell adhesion by the fast and sequential cell cleavages and associated mitotic rounding constantly remodelling and challenging the cell–cell contacts (Petridou *et al*, 2019). Interestingly, blastoderm fluidization is restricted to the blastoderm centre due to Wnt/PCP signalling being exclusively activated within the blastoderm margin (Makita *et al*, 1998; Ulrich *et al*, 2005; Witzel *et al*, 2006; Petridou *et al*, 2019) locally suppressing tissue fluidization there (Fig 2E) (Petridou *et al*, 2019). In contrast to the situation in *Xenopus* mesoderm, where Wnt/PCP signalling controls mesoderm tissue rigidity by increasing cell density (Barriga *et al*, 2018), tissue fluidization in the zebrafish blastoderm margin is thought to be blocked by Wnt/PCP signalling increasing cortical tension and cell–cell contact formation (Petridou *et al*, 2019) (Fig 2D and E). Interestingly, a similar spatial pattern of tissue fluidization was recently proposed to occur in the epiblast of the gastrulating chick embryo and to be essential for proper epiblast morphogenesis (preprint: Saadaoui *et al*, 2018). Reminiscent of the situation in the zebrafish blastoderm, this fluidization of the epiblast tissue also depends on cell divisions (Firmino *et al*, 2016; preprint: Saadaoui *et al*, 2018). Moreover, a contractile supra‐cellular actomyosin cable (a cable‐like arrangement of filamentous actomyosin spanning over several cell diameters) is formed at the marginal region of the epiblast, demarcating the region of tissue fluidization (preprint: Saadaoui *et al*, 2018) and overlapping in space where Wnt/PCP components are expressed within the gastrula (Voiculescu *et al*, 2007). This points to the intriguing possibility that Wnt/PCP signalling might function as an evolutionary conserved regulator of tissue viscosity/fluidity, and that this function is critical for proper tissue morphogenesis.

Besides Wnt/PCP signalling, other signalling pathways were proposed to control tissue fluidity in development. For instance, somite formation and anterior–posterior axis elongation in zebrafish depends on the movement of mesoderm progenitor cells through the mesoderm progenitor zone (MPZ) into the PSM (Fig 2F) (Kimelman, 2016). Using FDs to measure tissue yield stress, the MPZ was found to display low‐yield stress with cells undergoing extensive intermixing, while the PSM shows larger yield stress with little cell intermixing (Mongera *et al*, 2018) (Fig 2F). Moreover, model simulations suggested that the anterior–posterior expansion of the MPZ and PSM can be explained by the maturing PSM exhibiting a rigid state, which can support posterior elongation (Mongera *et al*, 2018). Yet, although several molecular components have been suggested to be important for MPZ and PSM elongation, it remains unclear how the above described rheological pattern is determined (Lawton *et al*, 2013). A potential key player is N‐cadherin, since N‐cadherin mutant fish display reduced elongation speeds and reduced yield stresses of the PSM (Mongera *et al*, 2018). However, whether N‐cadherin is indeed important for determining the rheology of the MPZ and/or PSM is still unclear (Lawton *et al*, 2013; Serwane *et al*, 2017; Mongera *et al*, 2018). In addition, canonical Wnt and FGF signalling were shown to be important for cell flows within the trunk and tail during zebrafish body axis extension, although they appear to function predominantly outside of the PSM where tissue rigidity is high (Lawton *et al*, 2013). In contrast, FGF/MAPK signalling during avian body axis elongation was clearly demonstrated to induce a posterior‐to‐anterior random cell motility gradient within the PSM. Further experimental and theoretical analyses suggested that this gradient of random motility results in a gradient of cell density within the PSM opposite to the cell motility gradient and triggers directional movement of cells towards the posterior end of the PSM, thereby driving body axis elongation (Bénazéraf *et al*, 2010).

It is becoming increasingly clear that signalling pathways affecting distinct cellular properties determining tissue architecture, such as cell packing, motion and adhesion, are “priming” certain regions within the developing organism to undergo changes in tissue rheology. Yet, how these cellular properties can influence the material phase of the tissue is not fully understood. In some cases, certain cell properties appear to linearly scale with the material property of the tissue (Shawky & Davidson, 2015; Barriga *et al*, 2018), while in other cases, small changes in cell properties can lead to drastic changes in the material state of the tissue akin a tissue phase transition (Mongera *et al*, 2018; Petridou *et al*, 2019). Phase transitions have been described in several biological systems, from macromolecules to cells and tissues. However, whether they occur within the developing embryo still needs to be clearly demonstrated. In the following section, we discuss the physical basis of phase transitions and the current indications of their occurrence in developing tissues.

## Occurrence and function of potential phase transitions in development

**Figure 3:**
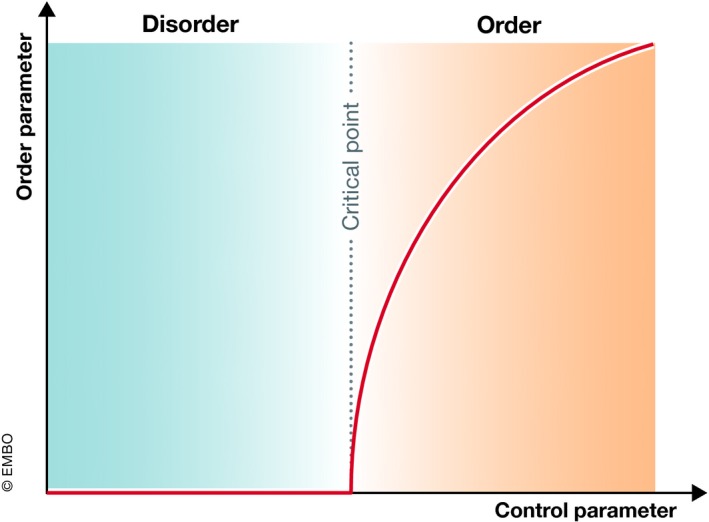
Phase diagram of an order–disorder phase transition When a large system undergoes a phase transition, at the critical point (dashed line), which corresponds to a certain value of the control parameter of the system (e.g. density, cell–cell adhesion, temperature), the order parameter (e.g. collective motion, rigid cluster size, magnetism) undergoes a sharp step (discontinuity) and diverges. The appearance or disappearance of the order parameter defines the ordered (e.g. solid‐like materials) or disordered (e.g. fluid‐like materials) states of the system, respectively.

Phase transitions describe the abrupt changes between the different states of matter and, more generally, the emergence of order in various systems, ranging from magnetism to crystallization (Fleury, 1981) (Glossary). The phase transition between order and disorder depends on several thermodynamic variables of the system, i.e. energy, temperature and entropy. At the phase transition point, the system exhibits key characteristics, such as criticality and *universality* (Glossary). Criticality refers to the existence of a critical point at which the system changes its phase (Fig 3). This critical point corresponds to a specific value of a control parameter*,* which once it is reached causes a discontinuous change in an *order parameter* (Fig 3 and Glossary). At the critical point, the material exhibits universality, meaning that the closer the control parameter is to its critical value, then the order parameter is less sensitive to the details of the system, thereby sharing specific common characteristics with other materials at the phase transition point. Other properties that a system may exhibit at the transition point is gain or loss of symmetry, e.g. during crystallization, and *scale invariance*, the presence of *power laws* for the divergence of thermodynamic properties (Glossary). Biological samples share the above characteristics of phase transitions; however, since they are not at thermal equilibrium, they do not undergo “true” phase transitions. Still, biological tissues exhibit distinct non‐equilibrium stationary phase states and under certain circumstances can undergo transitions between these states that mathematically can be approximated as equilibrium phase transitions. Such phase transitions in tissues include *solidification*,* jamming* or rigidity transition (fluid‐to‐solid phase transitions) and fluidization (solid‐to‐fluid phase transitions) (Glossary).

One influential formulation of phase transitions in active living matter is the Vicsek's flocking particle model or Vicsek's kinetic transition. Here, particles are assumed to have velocities that tend to align with the average velocity of their neighbours and display collective motion once reaching a critical density (Vicsek *et al*, 1995), a phenomenon typically observed in flocks of birds and schools of fish (Ballerini *et al*, 2008; Cavagna *et al*, 2010). Similar density‐dependent phase transitions were also experimentally observed in biological samples at the microscale (macromolecular networks) and mesoscale (cell assemblies). At the microscale, for instance, reconstituted actin and microtubule networks exhibit ordered transitions depending on the concentration of the monomers and/or density of the crosslinkers/motors, accompanied by self‐organization of the filaments in coherently moving structures such as clusters, swarms and swirls (Gardel *et al*, 2004; Schaller *et al*, 2010; Sanchez *et al*, 2012). At the meso‐ or cell scale, keratocytes, upon reaching a critical density, were found to undergo a kinetic phase transition from a disordered phase of random motion to an ordered phase of collective motion (Szabó *et al*, 2006). Control parameters other than cell density, such as cell–cell adhesion, cortical tension (Angelini *et al*, 2011; Bi *et al*, 2015, 2016) and geometry (Yang *et al*, 2017; Atia *et al*, 2018; Merkel & Manning, 2018), were also theoretically predicted to trigger fluid‐to‐solid phase transitions in cell assemblies once reaching a critical value. While the specific function of those different control parameters in phase transitions is not yet fully understood, the simple notion that the emergence of collective behaviours in living matter through phase transitions displays universality and scale invariance is highly intriguing. It implies that through phase transitions we can understand large‐scale emergent properties involved in tissue growth, spreading and folding.

One example where applying the concept of phase transitions has helped in understanding tissue behaviour is EMT during tissue spreading. By studying cell behaviour of 3D spheroidal aggregates placed on a solid and adhesive substrate, it was observed that over time the 3D cluster starts to spread on the substrate forming a 2D layer with cells individually leaving the cluster, a hallmark of EMT (Douezan *et al*, 2011). This process was further described by a *wetting* behaviour depending on the levels of E‐cadherin, where highly cohesive aggregates are in a liquid state, while in weakly cohesive aggregates, cells escape and undergo a liquid‐to‐gas transition (Douezan *et al*, 2011) (Glossary). The reverse transition, also called de‐wetting, was found in epithelial 2D monolayers, where increasing the level of E‐cadherin triggers a sudden morphological transition into 3D clusters (Pérez‐González *et al*, 2019). Theoretical modelling suggested that this de‐wetting transition is triggered at a critical point of cell contractility regulated by the E‐cadherin levels, and cell–substrate adhesion energy regulated by substrate ligand density and monolayer size (Pérez‐González *et al*, 2019). Understanding tissue spreading as a phase transition‐mediated process may also be important for understanding embryo morphogenesis. For instance, it was recently proposed that the spreading of the blastoderm over the yolk cell during zebrafish gastrulation can be—at least to some degree—described with wetting laws (Wallmeyer *et al*, 2018).

However, clearly describing such phase transitions at the organismal level is still challenging, since groups of cells within the organism are not purely self‐organizing through short‐range cell–cell interactions as for instance proposed in the flocking model, but they are rather exposed to external biochemical and mechanical signals that might introduce heterogeneity to the system. Consequently, whether and how embryonic tissues can undergo phase transitions is still unclear. That said, there is both theoretical and experimental evidence that embryonic tissues might display characteristics of *glassy materials* (i.e. sub‐diffusive trajectories and caging behaviour, Glossary) and might reside close to a phase (glass) transition point (Schoetz *et al*, 2013). This implies that small changes to a critical cellular control parameter could in principle generate an abrupt change in the viscoelastic property or order of the tissue. This notion seems to be supported by recent findings in zebrafish and chicken (described in more detail above) of a fluid‐to‐solid jamming transition in the PSM during body axis elongation (Bénazéraf *et al*, 2010; Mongera *et al*, 2018) and a fluidity transition in the blastoderm at the onset of zebrafish gastrulation (Petridou *et al*, 2019). The critical control parameters triggering these potential transitions appear to be quite similar between these different processes with the volume of extracellular spaces between cells and rate of random cell motion determining the jamming transition in the PSM, and the level of cell cohesion determining blastoderm fluidization. However, in order to clearly show that these processes indeed are phase transitions, a universal law addressing their criticality and universality still needs to be formulated.

## Discussion and outlook

Embryonic development is achieved by the spatial and temporal coordination of multiple interdependent events such as localized gene expression, morphogen gradient formation and perception, mechanical force generation, sensing and transduction, and viscoelastic dissipation of cells and tissues (Tabata & Takei, 2004; Farge, 2011; Heisenberg & Bellaïche, 2013; Petridou *et al*, 2017; Barriga & Mayor, 2018). Chemical and mechanical signalling also influence each other, enabling the generation of positive and negative feedback loops between the two (Chan *et al*, 2017; Kim *et al*, 2018; Hannezo & Heisenberg, 2019). Such mechanochemical feedback loops can act at the same scale or across different scales (molecular, cellular, tissue/organ), enabling the system to adapt and self‐organize effectively in response to micro‐ and/or mesoscale stimuli (Paluch, 2018). Yet, comparatively little is still known about how such mechanochemical feedback loops are influenced by tissue rheology, and how tissue rheology is controlled in development.

Intriguingly, the major regulators of tissue rheology can be assigned to key modules of embryo development, such as cell divisions, cell motion, cell adhesion and morphogen signalling (Lawton *et al*, 2013; Firmino *et al*, 2016; Barriga *et al*, 2018; Mongera *et al*, 2018; Petridou *et al*, 2019). Amongst those, cell division, and in particular mitotic rounding and cytokinesis, was suggested to increase tissue fluidity by promoting junctional remodelling (Ranft *et al*, 2010; Firmino *et al*, 2016; Petridou *et al*, 2019). Conversely, morphogen signalling, and specifically the Wnt/PCP pathway, was proposed to increase tissue rigidity by promoting junctional stability and cell density (Barriga *et al*, 2018; Petridou *et al*, 2019). Whether junctional stability and cell density are controlled by the same effector mechanisms or independently is not yet clear. Generally, to attribute the regulation of tissue rheological properties to certain structural or cellular features is still challenging, as these features are often interdependent, making functional assays rather difficult to interpret. For example, cell adhesion and contractility clearly influence each other (David *et al*, 2014; Pinheiro & Bellaïche, 2018), and modulating any of the two can also secondarily affect other processes, such as cell motion/intercalation, cell shape/geometry and cell density/packing, previously implicated in the control of tissue rheology. Systematic analysis of such features alone and in combination, and the development of theoretical models from statistical physics to, e.g., simulate network rigidity (i.e. vertex Voronoi, self‐propelled, percolation theory) will be needed to elucidate the molecular and cellular control mechanisms determining tissue rheology (Szabó *et al*, 2006; Garcia *et al*, 2015; Alt *et al*, 2017; Alvarado *et al*, 2017). Furthermore, different cell and tissue types might be differently regulated in terms of their rheological properties, and it thus will be important to consider cell fate specification and differentiation factors when analysing the regulation of tissue rheology.

Another so far largely unexplored question is whether tissue rheological properties are not only controlled by mechanochemical signalling but also affect such signalling. Previous studies suggested that tissue viscoelasticity influences the length scale of mechanical signal propagation, such as cortical flows in C. elegans zygotes (Mayer *et al*, 2010), or stress‐induced cell shape change propagation across the *Drosophila* wing imaginal disc epithelium (Duda *et al*, 2019). However, whether and how tissues displaying nonuniform rheology affect the transduction of long‐range mechanical signals generated by, e.g., supra‐cellular actomyosin cables (Behrndt *et al*, 2012; Röper, 2013; preprint: Saadaoui *et al*, 2018; Shook *et al*, 2018) remains unclear. For instance, tissue regions displaying large rheological differences may be mechanically insulated/isolated from each other, thereby generating a force–transduction boundary between them (Rodriguez‐Franco *et al*, 2017). It is also conceivable that tissue rheology not only affects mechanical but also chemical signal propagation. For instance, recent theoretical work proposed that fluidic cell clusters displaying increased random cell motion might be more competent to respond to a chemical gradient, as re‐positioning of the highly competent cells within these clusters towards the source of the gradient/ligand might be facilitated by increased random motion (Camley & Rappel, 2017). Whether such predicted effect indeed occurs within the developing embryo, and more generally, how the interplay between tissue rheology and morphogen signalling functions in development still needs to be explored.

Questions also remain as to the underlying mechanisms by which a group of individual active self‐propelled entities within the developing organism is ordered into large‐scale functional structures and compartments from where collective‐ordered behaviours, such as tissue folding and spreading, emerge. Recent methodological advances (Box 1) have allowed experimentalists to monitor closely the physical properties of such active systems, providing increasingly detailed information about specific properties of the system components. This information can then further be processed with tools from statistical mechanics in order to link individuality to the emergence of order. Such approaches have led to the hypothesis that biological systems might often be at the edge of instability, i.e. close to the critical point of phase transition (for review, see Munoz 2017). When a system is close to such a critical point, it is able to transit between ordered and disordered states, explaining how order or disorder can spontaneously emerge. In contrast, when the system is far from the critical point, it will be robust against perturbations. In biological systems, there are multiple cases where spontaneous order emerges, for instance during brain neural regions–circuits synchronization (di Santo *et al*, 2018), axon propagation (Turing, 1950; Chialvo, 2010; Hesse & Gross, 2014), gap gene expression network in *Drosophila* (Krotov *et al*, 2014), expression of genes defining stem‐cell pluripotency (Ridden *et al*, 2015), expression of symmetry‐breaking genes in *Hydra* (Gamba *et al*, 2012), collective motion of animals or cells (Vicsek *et al*, 1995; Szabó *et al*, 2006; Ballerini *et al*, 2008; Cavagna *et al*, 2010; Löber *et al*, 2015) and long‐range signalling in bacterial communities (Larkin *et al*, 2018). However, direct evidence for the spontaneous emergence of order transitions in developing tissues is still lacking. This might be due to difficulties in reliably monitoring such phase transitions *in vivo*, as only very recently non‐invasive tools became available that allow measuring tissue rheology *in vivo* (Box 1). Moreover, the characteristics of phase transitions, such as power laws, discontinuities and divergences, can formally only appear in the infinite‐size thermodynamic limit. The finite nature of biological tissues thus requires finite‐size scaling methods and the use of scaling laws to analyse the existence of critical phenomena. Since true criticality does not exist in finite systems, one needs to search for proxies of criticality, such as a progressive transition instead of a discontinuity, where a peak in the defined order parameter exists that shows divergence (Brézin, 1986; Chomaz & Gulminelli, 2002).

Finally, it is still unresolved how the developing organism benefits from being positioned close to criticality. Why would a living system be fitter if it is critical? Being at the borderline of two alternative regimes, order/stability versus disorder/instability, was proposed to offer the optimal balance between adaptation and robustness, which allows functionality, growth and evolution (Hidalgo *et al*, 2013). Likewise, in development, key characteristics of embryonic development such as symmetry breaking and the emergence of order and complexity are strikingly similar to hallmarks of phase transitions. Yet, whether and how phase transitions will help in understanding tissue morphogenesis in development is still unclear. One possibility is that phase transitions could explain why embryos across species display such a remarkable conservation in the series of irreversible morphogenetic events occurring during gastrulation and organogenesis.

## Conflict of interest

The authors declare that they have no conflict of interest.
